# Can the prognostic nutritional index predict survival in patients with gastric and esophagogastric junction adenocarcinoma?

**DOI:** 10.3389/fonc.2026.1872975

**Published:** 2026-10-22

**Authors:** Mehmet Furkan Sağdıç, Burak Dinçer, Güldeniz Argun, Ahmet Karayiğit, Cihangir Özaslan

**Affiliations:** 1 Department of Surgical Oncology, Ankara Etlik City Hospital, Ankara, Türkiye; 2 Department of Surgical Oncology, University of Health Sciences Ankara Oncology Training and Research Hospital, Ankara, Türkiye; 3 Department of Anesthesiology and Reanimation, University of Health Sciences Ankara Oncology Training and Research Hospital, Ankara, Türkiye

**Keywords:** prognostic nutritional index, gastric adenocarcinoma, esophagogastric junction cancer, prognostic biomarker, survival analysis

## Abstract

**Background:**

This study aimed to evaluate the prognostic significance of the preoperative Prognostic Nutritional Index (PNI) for overall survival (OS) and disease-free survival (DFS) in patients with gastric and gastroesophageal junction adenocarcinoma.

**Methods:**

We retrospectively analyzed the data from 300 patients undergoing curative gastrectomy at Ankara Oncology Training and Research Hospital between January 2013 and 2021. We calculated PNI using the formula: PNI = (10 × serum albumin [g/dL]) + (0.005 × total lymphocyte count). Using receiver operating characteristic (ROC) curve analysis, we determined the optimal PNI cut-off value as 45.83 and subsequently stratified patients into two groups: those with PNI ≥ 45.83 and those with PNI < 45.83. We then evaluated overall survival (OS) and disease-free survival (DFS) using the Kaplan–Meier method.

**Results:**

Our findings showed that patients with PNI < 45.83 had significantly shorter OS and DFS compared to those with PNI ≥ 45.83 (OS: 42 months vs. 90 months; DFS: 39 months vs. 84 months; p < 0.001). The multivariate analysis demonstrated low PNI, advanced T-stage, a higher number of metastatic lymph nodes, and Bormann type 3 tumors to be independent negative prognostic factors for both OS and DFS.

**Conclusions:**

Low preoperative PNI was associated with poorer OS and DFS in patients with gastric and gastroesophageal junction adenocarcinoma. PNI may contribute to preoperative risk assessment; however, prospective validation is required before its routine clinical use.

## 1 Introduction

Gastric cancer remains one of the malignancies with high incidence and mortality rates worldwide. About one million new cases are diagnosed each year, and it accounts for more than 800,000 deaths (1). As most patients with gastric cancer are asymptomatic in the early stages, the disease is often diagnosed at an advanced stage. Despite advances in multidisciplinary treatment, survival outcomes remain suboptimal in patients with locally advanced gastric cancer, while the prognosis is particularly poor in metastatic disease (2). Therefore, identifying non-invasive and easily accessible prognostic markers that can help predict disease outcomes in clinical practice is of key importance.

Recent research has demonstrated a close association between the Prognostic Nutritional Index (PNI) and the prognosis of patients with gastric cancer and reported that a low preoperative PNI is linked to poorer OS and disease-free survival (DFS) (3–10). Onodera et al. introduced PNI and designated it as a prognostic indicator from serum albumin concentration and peripheral blood lymphocyte count (10 × albumin [g/dL] + 0.005 × lymphocyte count) to evaluate preoperative surgical complications (3).

We hypothesized that a simple parameter obtained from routine blood tests may contribute to preoperative risk assessment and prognostic evaluation. Therefore, this study aimed to evaluate the prognostic value of preoperative PNI in patients with gastric and gastroesophageal junction adenocarcinoma. Although several studies have investigated the prognostic value of PNI in gastric cancer, most available evidence originates from East Asian populations, and substantial heterogeneity exists regarding patient selection, tumor location, treatment strategies, and optimal PNI cut-off values. Moreover, relatively limited data are available from Western populations including both gastric and gastroesophageal junction adenocarcinoma patients undergoing curative surgery. Therefore, additional validation in different clinical settings remains important.

## 2 Materials and methods

We retrospectively analyzed the preoperative and follow-up data from 300 patients with gastric or gastroesophageal junction adenocarcinoma who underwent surgery at Ankara Oncology Training and Research Hospital between January 2013 and 2021. Patients undergoing curative gastrectomy were included in the study, whereas we excluded those with metastatic disease, palliative or completion gastrectomy, non-adenocarcinoma pathologies, emergency surgeries, systemic diseases that could cause hypoalbuminemia (e.g., liver failure or nephrotic syndrome), concomitant hematologic or autoimmune disorders, incomplete medical records, or missing follow- up data. The patient selection process is shown in **Figure 1**. Patients with gastric adenocarcinoma and adenocarcinoma of the gastroesophageal junction were analyzed together because both malignancies share similar histopathological characteristics, are managed according to comparable curative surgical principles and perioperative treatment strategies and were treated within the same institutional multidisciplinary framework. Moreover, the primary objective of this study was to evaluate the prognostic performance of the Prognostic Nutritional Index (PNI), a systemic nutritional and immunological biomarker whose prognostic relevance is not specific to tumor location. The Research Ethics Committee of the University of Health Sciences, Ankara Oncology Training and Research Hospital granted ethical approval to our study (2024-07/104).

**Figure 1:**
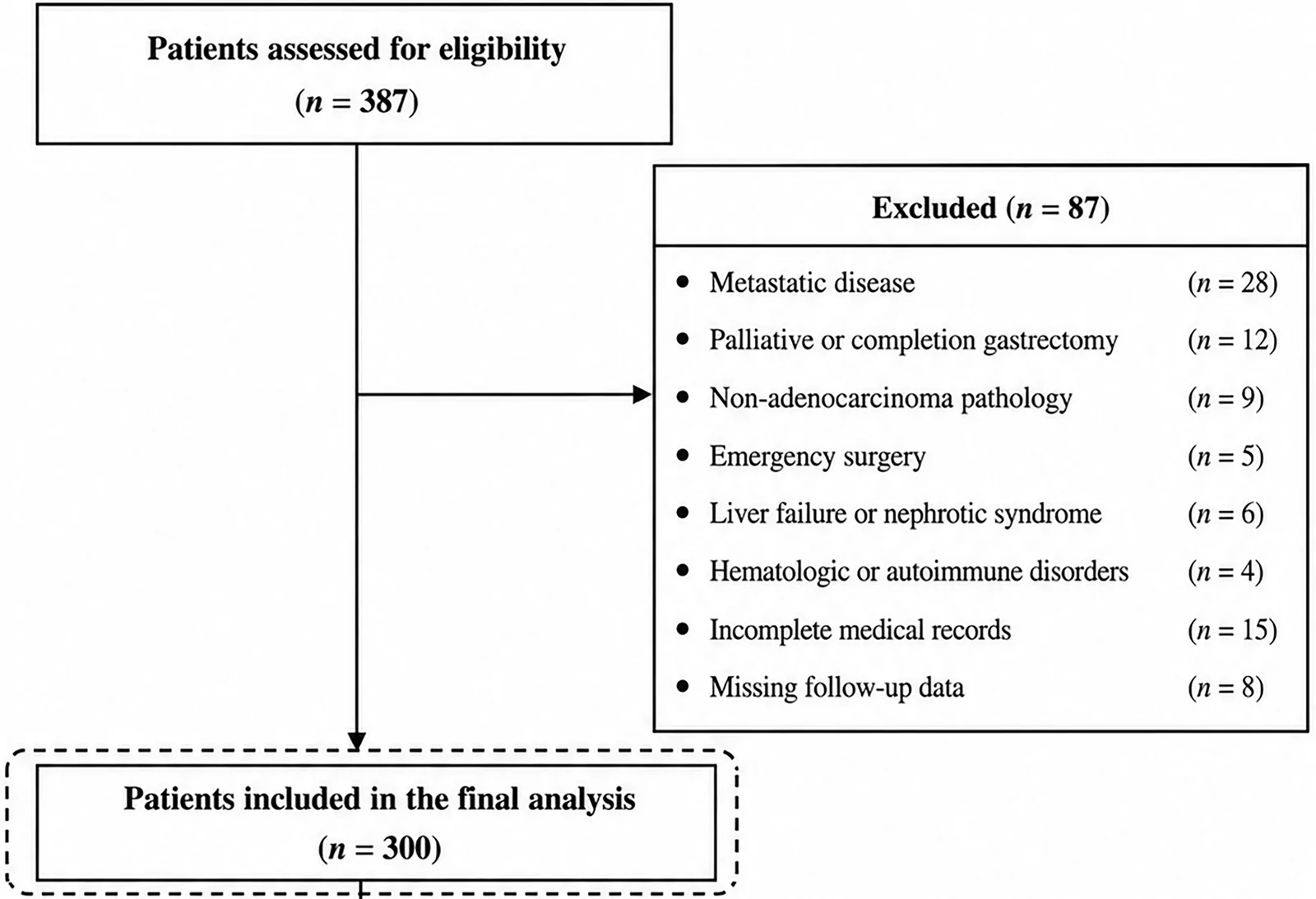
Flowchart of patient selection.

Through a retrospective review of the hospital database and patient records, we collected data on age, sex, date of surgery, date of last follow-up, presence of distant metastasis, presence and timing of local recurrence, site and timing of distant metastasis, follow-up duration, tumor size, perineural invasion, lymphovascular invasion, histological type and Laurén–WHO classification, macroscopic (Bormann) type, differentiation status, depth of invasion, disease stage, tumor location, number of dissected lymph nodes, lymph node metastasis status, number of metastatic lymph nodes, additional organ resection, type of surgery, neoadjuvant chemotherapy status, and adjuvant chemotherapy or radiotherapy status.

For the PNI assessment, we considered preoperative blood samples and laboratory measurements taken for anesthesia preparation, up to 2 weeks prior to surgery. If multiple samples were available within this timeframe, we considered those closest to surgery. All patients underwent upper endoscopy, biopsy, and computed tomography. Staging was based on clinical and radiological findings. We calculated PNI upon the following equation: [(10 × serum albumin (g/dL)) + (0.005 ×total lymphocyte count)].

We determined optimal cut-off values for PNI through receiver operating characteristic (ROC) curve analysis. The ROC analysis was performed based on survival status at the last follow- up (alive vs deceased) to determine the optimal cut-off value for PNI. The optimal threshold was identified using the maximum Youden index. The point showing the highest Youden index was identified as the optimal threshold. Patients were subsequently stratified according to these cut-off values. We then assessed OS and DFS. Overall survival was defined as the interval from the date of surgery to death from any cause. We defined DFS as the period from surgery to the first documented recurrence or mortality from any cause. Clinical, radiological, or endoscopic findings were used to confirm disease recurrence. Cumulative survival curves for OS and DFS were generated using the Kaplan–Meier (KM) method, and differences between groups were evaluated with the log-rank test. We calculated hazard ratios (HR) and corresponding 95% confidence intervals (CI) using the Cox proportional hazards regression model. We performed univariate and multivariate analyses. Variables with p < 0.05 in univariate analysis were included in the multivariate Cox regression model. SPSS version 25.0 (IBM Corporation, Armonk, NY, USA) was used for all statistical analyses. A p-value of < 0.05 was considered statistically significant.

## 3 Results

We analyzed the data from a total of 300 patients. To categorize patients, we determined the optimal cut-off PNI value. The median follow-up duration for the entire cohort was 23.5 months (mean: 43.2 ± 42.2 months, range: 0–154 months). The area under the ROC curve (AUC) was 0.707 (95% CI: 0.647–0.768; p < 0.001; **Figure 2**). This value indicates modest discriminatory ability. The Youden index reached its maximum when the PNI was 45.83. Accordingly, patients were distributed into two groups: PNI ≥ 45.83 (n = 146) and PNI <45.83 (n = 154).

**Figure 2:**
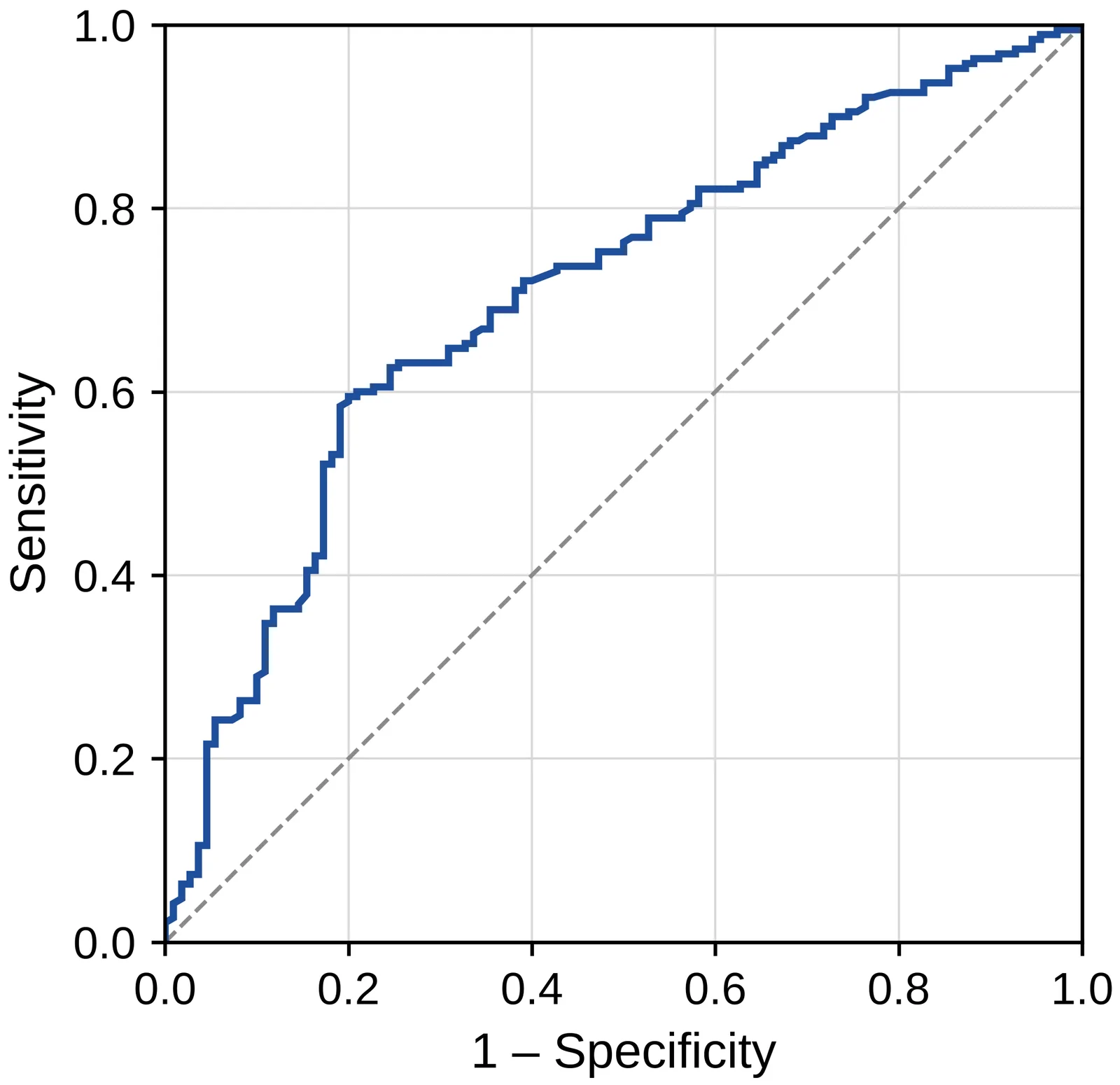
ROC curve of the preoperative prognostic nutritional index (PNI) for overall survival. The optimal cut-off was 45.83 (AUC = 0.707; 95% CI: 0.647–0.768; p < 0.001), indicating modest discriminatory ability.

Of patients, 146 were classified into the PNI ≥ 45.83 group, and 154 into the PNI < 45.83 group. The mean age was 61.7 ± 11.5 years (median = 63, range = 35–86 years) in the PNI ≥ 45.83 group and 66.7 ± 11.7 years (median = 66, range = 32–90 years) in the PNI < 45.83 group (p < 0.001). The groups were similar by sex (male/female = 71%/29% vs. 73%/27%, p = 0.773). Regarding surgical approach, open surgery was performed in 86% of patients with PNI ≥ 45.83 and 91% of those with PNI < 45.83 (p = 0.153). The rates of neoadjuvant therapy were comparable between the groups (14% vs. 13%, p = 0.749). According to Bormann classification, the distribution of macroscopic tumor types was as follows: 26% (type 1), 34% (type 2), 35% (type 3), and 5% (type 4) in the PNI ≥ 45.83 group, and 25% (type 1), 38% (type 2), 31% (type 3), and 6% (type 4) in the PNI < 45.83 group (p = 0.832). When it comes to the Laurén classification, the intestinal, diffuse, and mixed types were detected in 61%, 34%, and 5% of patients with PNI ≥ 45.83, and in 60%, 35%, and 5% of those with PNI < 45.83, respectively (p = 0.951). Tumor location was proximal in 31%, middle in 30%, distal in 35%, and diffuse in 4% of the PNI ≥ 45.83 group, while these rates were 34%, 25%, 39%, and 2% in the PNI < 45.83 group, respectively (p = 0.602). Adenocarcinoma, signet ring cell, poorly cohesive, and mixed types were detected in 66%, 7%, 20%, and 7% of the PNI ≥ 45.83 group and in 64%, 10%, 23%, and 3% of the PNI < 45.83 group, respectively (p = 0.355). Perineural invasion was present in 35% of patients with PNI ≥ 45.83 and 39% with PNI < 45.83 (p =0.503). Lymphatic invasion was significantly more prevalent in the PNI < 45.83 group (51% vs. 37%, p = 0.017), as was venous invasion (49% vs. 36%, p = 0.030). In terms of tumor depth, 36% of patients in the PNI ≥ 45.83 group were in T1–T2 stages, whereas 64% were in T3–T4. These rates were 19% and 81% in the PNI < 45.83 group, respectively (p = 0.001). Lymph node negativity and positivity were 30% and 70% in the PNI ≥ 45.83 group, respectively. They were detected in 22% and 78% of patients in the PNI < 45.83 group, respectively (p = 0.112). The mean number of dissected lymph nodes was 21.1 ± 11 (median = 20) in the PNI ≥ 45.83 group and 21.6 ± 10.9 (median =20.5) in the PNI < 45.83 group (p = 0.660). The mean number of metastatic lymph nodes was significantly higher in the PNI < 45.83 group (8.06 ± 8.64; median = 6) compared to the PNI ≥ 45.83 group (4.9 ± 6.95; median = 2; p < 0.001). Patients’ clinicopathological characteristics by PNI are presented in **Table 1**.

**Table 1:** Patients’ clinicopathological characteristics.

Clinicopathological Characteristics	PNI ≥45.83	PNI <45.83	p-value
	n=146 (%)	n=154 (%)	
Age Mean Median	61.7±11.5 63 (35-86)	66.7±11.7 66 (32-90)	<0.001*
Sex Male Female	104 (71) 42 (29)	112 (73) 42 (27)	0.773
Surgery type Open Laparoscopic	125 (86) 21 (14)	140 (91) 14 (9)	0.153
Comorbidities Absence Presence	29 (20) 117 (80)	23 (15) 131 (85)	0.330
Neoadjuvant therapy No Yes	127 (87) 19 (13)	132 (86) 22 (14)	0.749
Macroscopic type (Bormann) Type 1 (polypoid-vegetative) Type 2 (ulcerative-vegetative) Type 3 (ulcerative-infiltrative) Type 4 (linitis)	38 (26) 50 (34) 51 (35) 7 (5)	38 (25) 59 (38) 48 (31) 9 (6)	0.832
Tumor localization Proximal Middle Distal Diffuse	45 (31) 44 (30) 51 (35) 6 (4)	52 (34) 38 (25) 60 (39) 4 (2)	0.602
Histologic type (Laurén) Intestinal	89 (61)	91 (59)	0.951

The mean OS for the entire cohort was 65.7 months (95% CI, 58.1–73.3), with a median OS of 24 months (95% CI, 17.4–30.5; **Figure 3**). The mean DFS was 62.0 months (95% CI, 54.3–69.8), and the median DFS was 18 months (95% CI, 12.5–23.5; **Figure 4**).

**Figure 3:**
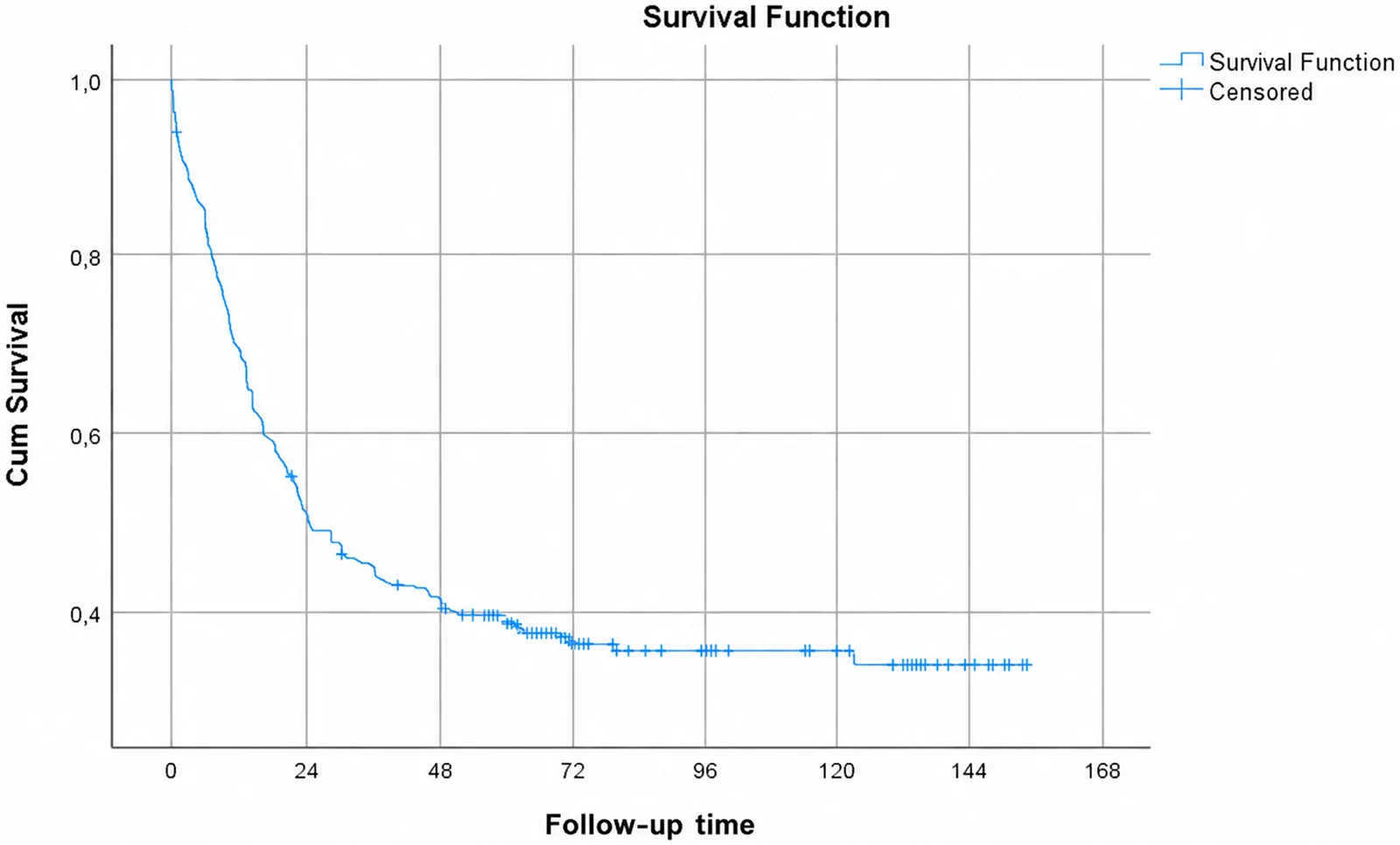
Overall survival (OS) Kaplan-Meier curve.

**Figure 4:**
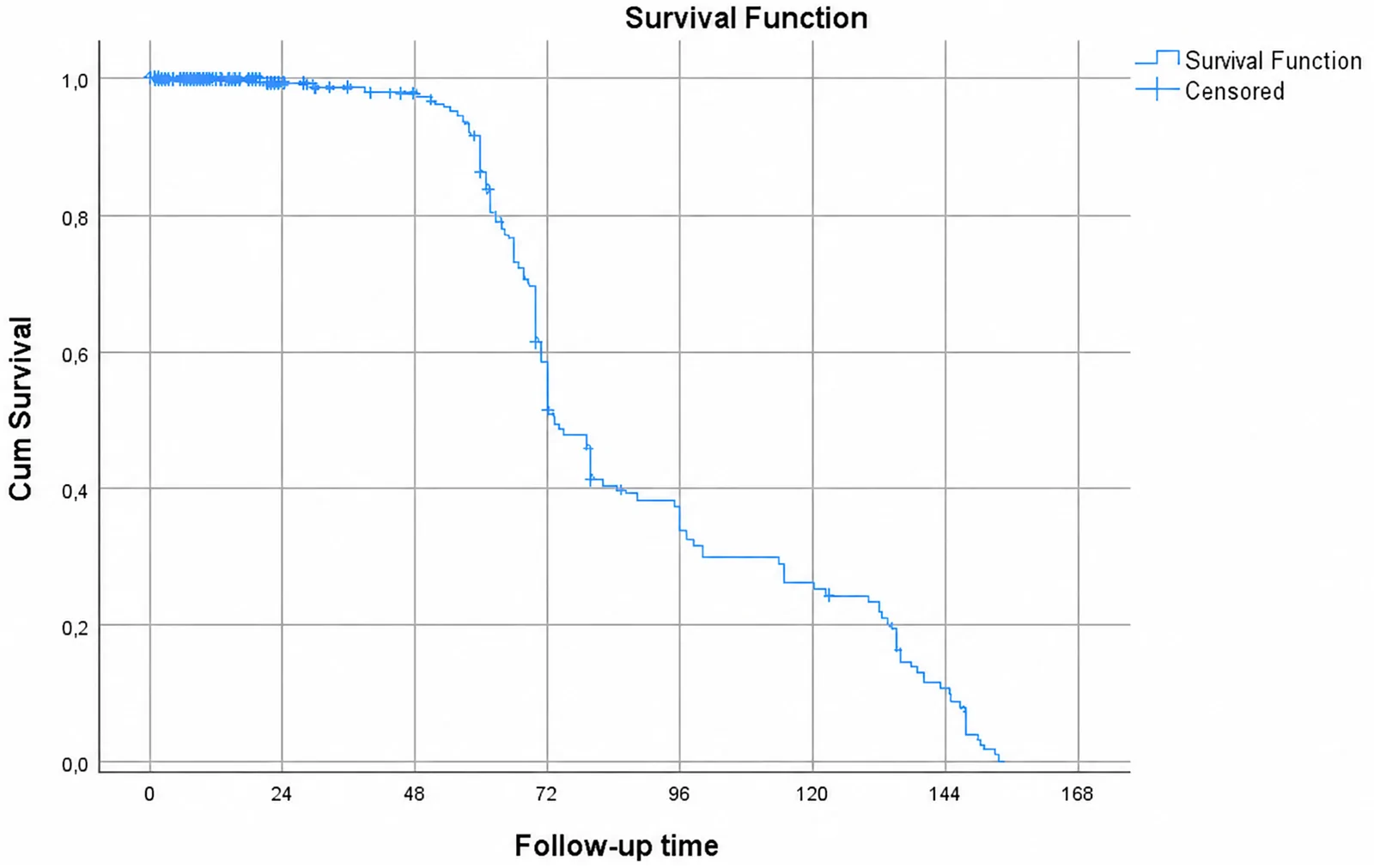
Disease-free survival (DFS) Kaplan-Meier curve.

OS was significantly shorter in patients with low PNI compared to those with high PNI (p < 0.001; 42 months vs. 90 months; **Figure 5**). Similarly, DFS was significantly shorter in the low PNI group (p < 0.001; 39 months vs. 84 months; **Figure 6**). The PNI ≥ 45.83 group demonstrated a mean OS of 90.2 ± 5.8 months (95% CI, 78.8–101.6). The median OS was not reached, as the estimate was limited by the longest observed survival time due to a high proportion of censored cases. In contrast, patients with PNI < 45.83 demonstrated a mean OS of 42.0 ± 4.4 months (95% CI, 33.4–50.7) and a median OS of 15.6 ± 2.5 months (95% CI, 10.7–20.5), also limited by censoring.

**Figure 5:**
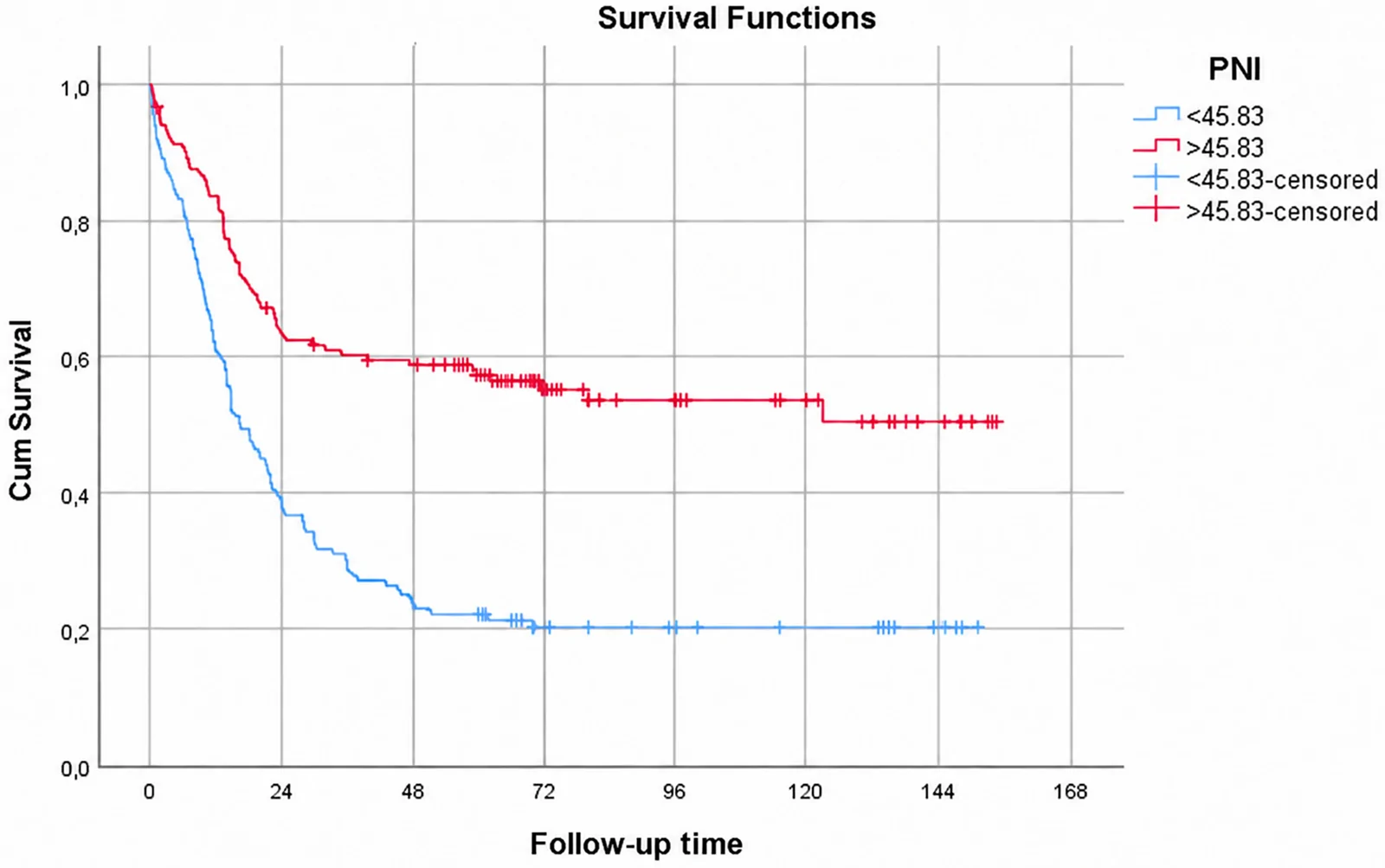
Overall survival (OS) by PNI groups.

**Figure 6:**
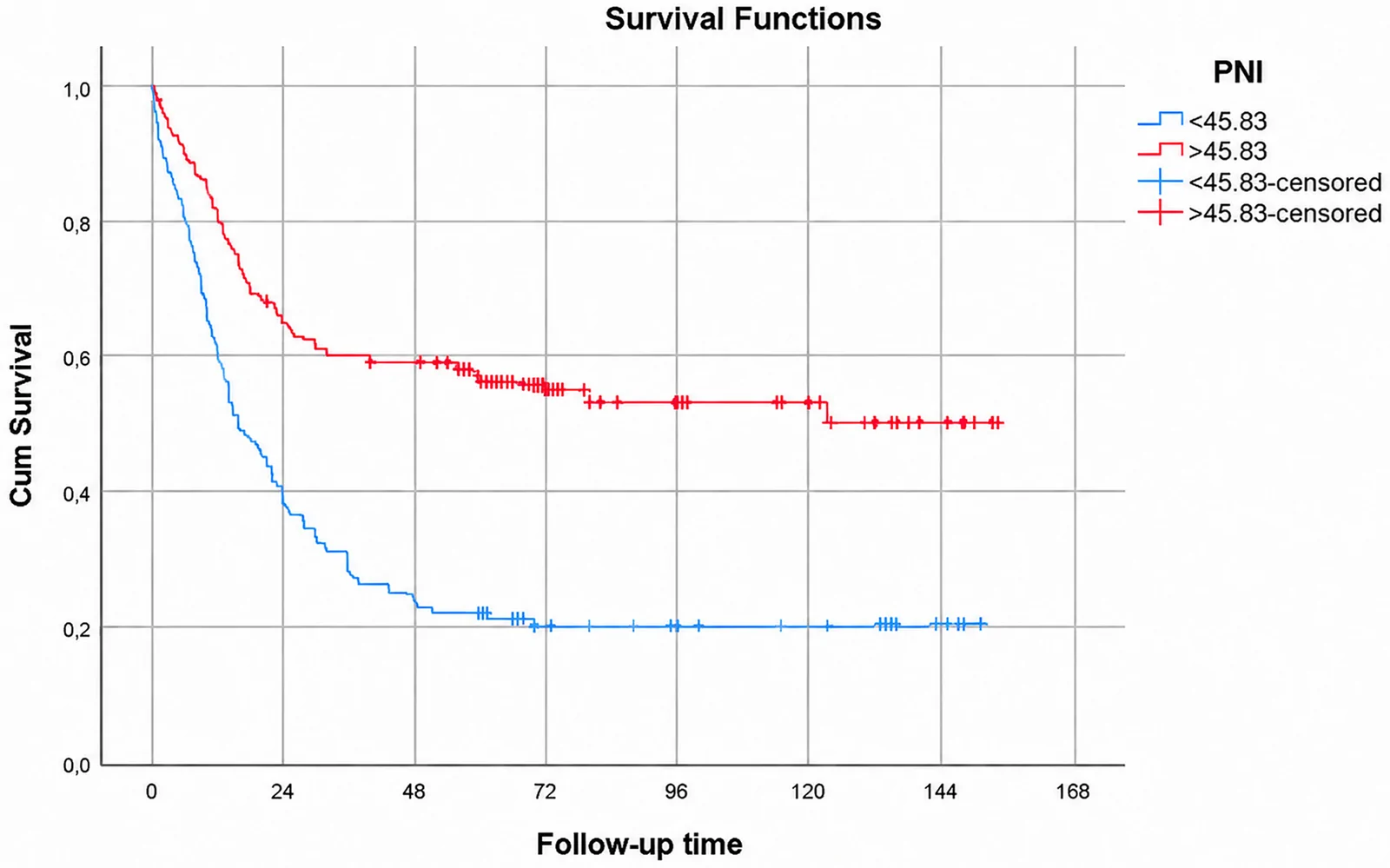
Disease-free survival (DFS) by PNI groups.

Patients with PNI ≥ 45.83 had a mean DFS of 85.0 ± 6.0 months (95% CI, 73.3–96.6). The median DFS was not reached, as the estimate was limited by the longest observed survival time due to a high proportion of censored cases. In contrast, patients with PNI < 45.83 had a mean DFS of 39.8 ± 4.5 months (95% CI, 31.0–48.6) and a median DFS of 12.0 ± 1.3 months (95% CI, 9.4–14.6), also limited by censoring.

The univariate analysis for OS revealed PNI ≥ 45.83, perineural invasion, lymphatic invasion, venous invasion, T3–T4 tumor stage, number of metastatic lymph nodes, number of dissected lymph nodes, and Bormann type 3 tumors to be significant prognostic factors (p < 0.05). In the multivariate analysis, we identified PNI ≥ 45.83, T3–T4 stage, number of metastatic lymph nodes, and Bormann type 3 tumor type as independent prognostic factors negatively affecting OS (p < 0.05; **Table 2**).

**Table 2:** Univariate and multivariate analyses of the factors associated with OS.

	Univariate analysis			Multivariate analysis		
	Hazard ratio	CI 95%	p-value	Hazard ratio	CI 95%	p-value
Age	0.987	0.961-1.014	0.140			
Sex	1.255	0.828–1.903	0.285			
Surgery type	1.530	0.847–2.765	0.159			
Adjuvant chemotherapy	1.620	1.039–2.525	0.043			
Adjuvant radiotherapy	1.073	0.673–1.712	0.767			
PNI ≥ 45.83	0.398	0.295-0.538	<0.001	0.492	0.361-0.671	<0.001
Perineural invasion	2.063	1.546-2.753	<0.001			
Lymphatic invasion	2.363	1.769-3.160	<0.001			
Venous invasion	2.364	1.770-3.157	<0.001			
Pathological T3-4	4.507	2.879-7.057	<0.00 1	2.587	1.557-4.301	<0.001
Number of metastatic lymph nodes	1.052	1.039-1.066	<0.001	1.026	1.009-1.042	0.002
Number of dissected lymph nodes	1.019	1.006-1.031	0.003			
Macroscopic type (Bormann) Type 3	3.415	1.886-6.182	<0.001	2.59	1.393-4.816	0.003

In the univariate analysis for DFS, PNI ≥ 45.83, perineural and lymphatic invasion, venous invasion, T3–T4 tumor stage, number of metastatic and dissected lymph nodes, and Bormann type 3 tumors were significantly associated with DFS (p < 0.05). Multivariate analysis revealed that PNI > 45.83, T3–T4 stage, number of metastatic lymph nodes, and Bormann type 3 tumors independently predicted poorer DFS (p < 0.05; **Table 3**).

**Table 3:** Univariate and multivariate analyses of the factors associated with DFS.

	Univariate analysis			Multivariate analysis		
	Hazard ratio	CI 95%	p-value	Hazard ratio	CI 95%	p-value
Age	1.023	1.010–1.036	<0.00 1	1.019	1.003-1.035	0.017
Sex	1.267	0.918–1.747	0.150			
Surgery type	0.947	0.607–1.476	0.810			
Adjuvant chemotherapy	1.496	1.007–2.222	0.046			
Adjuvant radiotherapy	1.618	1.179–2.220	0.003			
PNI ≥ 45.83	0.436	0.325-0.586	<0.00 1	0.564	0.414-0.768	<0.001
Perineural invasion	2.059	1.548-2.738	<0.001			
Lymphatic invasion	2.471	1.855-3.292	<0.001			
Venous invasion	2.473	1.858-3.292	<0.001			
Pathological T3-4	4.223	2.722-6.551	<0.001	2.175	1.283-3.687	0.004
Number of metastatic lymph nodes	1.051	1.038-1.064	<0.001	1.03	1.009-1.051	0.004
Number of dissected lymph nodes	1.018	1.006-1.031	0.003			
Macroscopic type (Bormann) Type 3	2.873	1.594-5.177	<0.001	1.644	1.103-2.449	0.015

## 4 Discussion

In the present study, preoperative PNI was significantly associated with overall survival in patients with gastric and gastroesophageal junction adenocarcinoma undergoing curative surgery. Patients with a low PNI had significantly poorer overall survival and more frequently exhibited adverse pathological characteristics, including lymphatic invasion, venous invasion, advanced pathological T stage, and a higher number of metastatic lymph nodes. Moreover, PNI remained an independent prognostic factor in multivariate analysis, highlighting its potential value as a complementary marker for preoperative risk assessment.

The PNI is calculated using serum albumin levels and total lymphocyte count. Since both albumin and lymphocyte levels are routinely measured in preoperative laboratory tests, PNI calculation imposes no additional cost. Serum albumin serves as an indicator of nutritional status, and hypoalbuminemia was previously reported to be associated with cancer progression and poor survival outcomes across various malignancies (4–8). Lymphocytes, on the other hand, are immune cells that inhibit tumor cell proliferation through cytokine-mediated cytotoxic mechanisms; hence, reduced lymphocyte count may reflect immunosuppression or impaired immune function (9, 10).

Our findings suggest that a low PNI reflects not only impaired nutritional status but also a more aggressive disease profile. In the present study, patients with low PNI more frequently exhibited adverse pathological characteristics, including higher rates of lymphatic invasion, venous invasion, advanced pathological T stage, and a greater number of metastatic lymph nodes. These findings suggest that a reduced PNI may be associated with increased tumor burden and more advanced disease, which may partly explain the poorer survival outcomes observed in this group. Therefore, PNI appears to represent a composite biomarker reflecting both host nutritional–immune status and tumor aggressiveness.

The PNI is an established, reliable prognostic indicator in various malignancies, including gastrointestinal, lung, breast, gynecological, and genitourinary cancers (11–18). In gastric cancer, it has recently attracted attention as a non-invasive and easily calculable prognostic marker. Previous research identified PNI as an independent risk factor for survival (19–29). However, some studies failed to confirm this finding. For instance, Toyokawa et al. examined 225 patients with Stage III gastric adenocarcinoma and found no significant association between OS and PNI (25). Similarly, Hirahara et al. included 368 patients in their study and reported that although PNI was significantly associated with OS in univariate analysis, this association was not maintained in multivariate analysis (30).

On the other hand, large-scale studies from South Korea, among the most comprehensive in the literature, demonstrated the prognostic value of PNI. Jung et al. analyzed 8,014 patients with stage T1N0-1M0 and T2–3N0M0 gastric cancer, while Lee et al. evaluated 7,781 patients; both studies identified PNI as an independent risk factor for survival (27, 31). Similarly, a 2024 meta-analysis by Deng et al. with 23,756 patients reported that patients with low PNI had significantly shorter OS, recurrence-free survival, and cancer-specific survival compared with those with high PNI. The cut-off values in the meta-analysis ranged from 40 to 52 (32). In their meta-analysis, Li et al. reported the cut-off values varying between 40 and 51 (33).

In our study, we calculated the cut-off PNI value as 45.83 and found that PNI was an independent prognostic factor for both OS and DFS. Patients with low PNI had significantly shorter OS (p < 0.001; 42 months vs. 90 months) and DFS (p< 0.001; 39 months vs. 84 months) compared with those with high PNI. Consistent with the literature, our findings confirmed that lower PNI is associated with poorer OS and DFS. Although our cut-off value of 45.83 aligns with the range reported in previous studies, the wide variation in cut-off thresholds across populations limits the routine clinical implementation of PNI as a standardized prognostic biomarker. Nonetheless, defining a population- and center-specific cut-off value could support clinical decision-making during the treatment process. If standardized, PNI could serve as a valuable decision-support tool for preoperative risk assessment, personalized treatment planning, prognosis estimation, and postoperative follow-up. Although PNI was significantly associated with survival outcomes, its discriminatory ability was modest (AUC = 0.707). Therefore, PNI should not be considered a stand-alone prognostic tool but rather a complementary parameter that may be interpreted alongside established clinicopathological factors.

From a clinical perspective, preoperative PNI may help identify patients at increased nutritional risk before surgery. Patients with a low PNI may benefit from nutritional assessment and individualized perioperative nutritional support. In addition, these patients may benefit from closer perioperative monitoring and careful optimization of their overall clinical condition before surgery. However, the present study does not provide evidence that PNI should be used to modify surgical or oncological treatment strategies. Rather, PNI should be considered a complementary tool for preoperative risk assessment, and prospective interventional studies are required to determine whether targeted nutritional or perioperative interventions based on PNI can improve clinical outcomes. However, our study was not designed to evaluate whether improving PNI leads to better oncological outcomes. Therefore, prospective studies are needed to determine whether preoperative nutritional interventions can improve long-term survival.

Our study also adds to the existing evidence in several important aspects. Unlike many previous studies that were predominantly conducted in East Asian populations, our cohort represents a real-world experience from a tertiary referral center and includes both gastric and gastroesophageal junction adenocarcinoma patients undergoing curative surgery. In addition, instead of adopting a previously reported threshold, we determined a population-specific optimal PNI cut-off value using ROC analysis. Furthermore, PNI remained an independent prognostic factor even after adjustment for established clinicopathological variables, supporting its potential clinical utility as a simple and practical tool for preoperative risk stratification. In addition, we found advanced T stage, number of metastatic lymph nodes, and Bormann type 3 tumor to be independent prognostic factors adversely affecting both OS and DFS, consistent with previous research (34–37).

In addition to PNI, we identified Borrmann type III tumors as an independent adverse prognostic factor for both overall and disease-free survival. The prognostic significance of the Borrmann classification has been evaluated in several large retrospective studies. A multicenter analysis including 3,966 patients demonstrated that Borrmann type was an independent prognostic factor after adjustment for tumor depth and nodal status, with significantly poorer survival in patients with type III and IV tumors than in those with type I and II tumors (38). Similarly, a cohort study of 2,092 patients reported that Borrmann type III and IV tumors were associated with larger tumor size, poorer differentiation, more advanced TNM stage, and significantly worse overall survival, and confirmed Borrmann classification as an independent predictor of prognosis following curative resection (39). Furthermore, another large study involving 2,604 patients showed that Borrmann classification remained an independent prognostic factor even after adjustment for vascular invasion and other clinicopathological variables, while patients with Borrmann type III tumors combined with vascular invasion had survival outcomes comparable to those with Borrmann type IV disease (40) The infiltrative-ulcerative growth pattern characteristic of Borrmann type III tumors may facilitate deeper wall invasion, lymphovascular dissemination, and nodal metastasis, thereby reflecting a more aggressive tumor biology. Therefore, our findings are consistent with the existing literature and further support the incorporation of macroscopic Borrmann classification into routine prognostic assessment of patients undergoing curative gastrectomy.

This study is presented with its few limitations. Due to its retrospective design, there may be a potential risk of selection bias. The PNI cutoff was derived using conventional ROC analysis based on binary survival status, which does not account for differences in follow-up duration or censored observations. A time-dependent ROC approach would have been methodologically more appropriate. Furthermore, the same cutoff was applied to the DFS analysis without independent optimization for this outcome. Therefore, the prognostic findings based on this cutoff should be considered exploratory and require validation using time-to-event–based methods in independent cohorts. In addition, the relatively short and variable follow-up duration limited the maturity of the survival data. Median OS was not reached in the high-PNI group; therefore, this finding should not be interpreted as a definitive estimate of long-term survival. The Kaplan–Meier estimates may have been influenced by a limited number of patients with prolonged follow-up and censored observations. Longer follow-up is required to confirm the observed survival differences between the PNI groups. The proportion of patients receiving neoadjuvant therapy was relatively low because the study included patients treated between 2013 and 2021, during which indications for neoadjuvant treatment evolved over time and clinical practice differed from current recommendations. In addition, neoadjuvant therapy may influence serum albumin and lymphocyte levels and consequently affect PNI values. Although the proportion of patients receiving neoadjuvant therapy was similar between the two study groups, this potential effect should be considered when interpreting our findings. Furthermore, the opposite-direction associations observed between adjuvant chemotherapy or radiotherapy and survival may reflect confounding by indication and treatment-selection bias and should therefore not be interpreted as causal treatment effects. In addition, the inconsistent inclusion of variables in the OS and DFS multivariable models represents a methodological limitation, and these findings should be interpreted with caution. Moreover, the generalizability of the findings from this single-center study may be limited. Nevertheless, its large patient cohort, the comprehensive analysis of detailed clinical and histopathological data, and the evaluation of a homogeneous patient group enhance the methodological robustness of the research. The use of preoperative data further contributes to the clinical relevance of the study by providing a framework with high applicability for pre-surgical risk stratification. Future prospective multicenter studies with larger patient populations are warranted to validate the optimal PNI cut-off and to determine whether perioperative nutritional optimization based on PNI can improve postoperative and long-term oncological outcomes. Such studies may further clarify the role of PNI as a complementary tool in preoperative risk assessment.

Overall, we found that the preoperatively calculated PNI was an independent prognostic marker for both OS and disease-free survival DFS in patients with gastric and gastroesophageal junction adenocarcinoma. Patients with low PNI values had markedly shorter survival durations compared to those with higher PNI scores. As a non-invasive and easily obtainable parameter derived from routine laboratory measurements, PNI may contribute to preoperative risk assessment and help identify patients who may benefit from further nutritional evaluation and perioperative optimization. However, prospective studies are required before PNI can be incorporated into routine clinical decision-making or used to guide individualized treatment strategies.

## Data Availability

The data analyzed in this study is subject to the following licenses/restrictions: Restrictions apply to the availability of these data due to ethical and legal considerations related to patient privacy. The datasets generated and/or analyzed during the current study are not publicly available but are available from the corresponding author on reasonable request, subject to approval by the institutional ethics committee. Requests to access these datasets should be directed to Mehmet Furkan Sağdıç e-mail: m.f.sagdic@gmail.com. Requests to access these datasets should be directed to m.f.sagdic@gmail.com.
